# Implant Survival, Adverse Events, and Bone Remodeling of Osseointegrated Percutaneous Implants for Transhumeral Amputees

**DOI:** 10.1007/s11999-014-3695-6

**Published:** 2014-05-31

**Authors:** Georgios Tsikandylakis, Örjan Berlin, Rickard Brånemark

**Affiliations:** Department of Orthopedics, Sahlgrenska University Hospital, 416 85 Gothenburg, Sweden

## Abstract

**Background:**

Osseointegrated percutaneous implants provide direct anchorage of the limb prosthesis to the residual limb. These implants have been used for the rehabilitation of transhumeral amputees in Sweden since 1995 using a two-stage surgical approach with a 6-month interval between the stages, but results on implant survival, adverse events, and radiologic signs of osseointegration and adaptive bone remodeling in transhumeral amputees treated with this method are still lacking.

**Questions/purposes:**

This study reports on 2- and 5-year implant survival, adverse events, and radiologic signs of osseointegration and bone remodeling in transhumeral amputees treated with osseointegrated prostheses.

**Methods:**

Between 1995 and 2010, we performed 18 primary osseointegrated percutaneous implants and two implant revisions in 18 transhumeral amputees; of those, 16 patients were available for followup at a minimum of 2 years (median, 8 years; range, 2–19 years). These include all transhumeral amputees who have received osseointegrated prostheses and represented approximately 20% of the all transhumeral amputees we evaluated for potential osseointegration during that time; general indications for this approach included transhumeral amputation resulting from trauma or tumor, inability to wear or severe problems wearing a conventional socket prosthesis, eg, very short residual limb, and compliant patients. Medical charts and plain radiographs were retrospectively evaluated.

**Results:**

The 2- and 5-year implant survival rates were 83% and 80%, respectively. Two primary and one revised implant failed and were removed because of early loosening. A fourth implant was partially removed because of ipsilateral shoulder osteoarthritis and subsequent arthrodesis. The most common adverse event was superficial infection of the skin penetration site (15 infections in five patients) followed by skin reactions of the skin penetration site (eight), incomplete fracture at the first surgery (eight), defective bony canal at the second surgery (three), avascular skin flap necrosis (three), and one deep implant infection. The most common radiologic finding was proximal trabecular buttressing (10 of 20 implants) followed by endosteal bone resorption and cancellization (seven of 20), cortical thinning (five of 20), and distal bone resorption (three of 20).

**Conclusions:**

The implant system presented a survivorship of 83% at 5 years and a 38% 5-year incidence of infectious complications related to the skin penetration site that were easily managed with nonoperative treatment, which make it a potentially attractive alternative to conventional socket arm prostheses. Osseointegrated arm prostheses have so far only been used in transhumeral amputations resulting from either trauma or tumor. Their use has not been tested and is therefore not recommended in transhumeral amputations resulting from vascular disease. This method could theoretically be superior to socket prostheses, especially in transhumeral amputees with very short residual humerus in which the suspension of a conventional prosthesis is difficult. Comparative studies are needed to support its potential superiority. Moreover, the radiological findings in this study need to be followed over time because some of them are of uncertain long-term clinical relevance.

**Level of Evidence:**

Level IV, case series. See Guidelines for Authors for a complete description of levels of evidence.

## Introduction

**Fig. 1:**
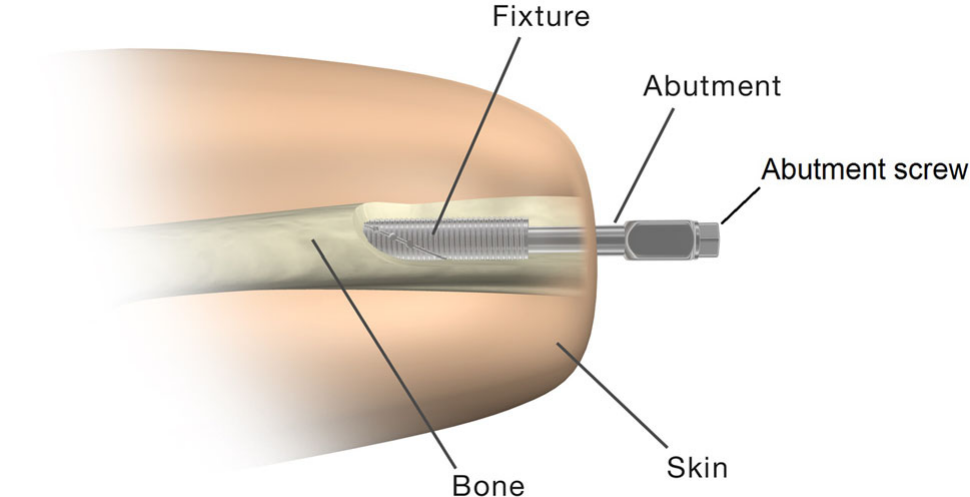
The percutaneous implant that was used in our study consists of three parts: the fixture, the abutment, and the abutment screw. Reproduced with permission and copyright © of the British Editorial Society of *Bone and Joint Surgery* [2].

It has been 19 years since the principle of osseointegration was implemented for the rehabilitation of transhumeral amputees in Sweden. This method allows direct anchorage of the prosthetic limb to the humeral bone using a threaded titanium implant (fixture), which is surgically attached into the residual bone at a first operation (S1) [4]. At a second operation (S2), a titanium extension (abutment) is inserted into the fixture and secured with an abutment screw (Fig. 1). The abutment penetrates the skin and serves as the anchoring point for the attachment of the prosthetic limb.

The idea of a bone-anchored implant penetrating the skin and coming in direct contact with the outer environment is challenging and often met with skepticism because the outer part of such an implant is inevitably contaminated by the skin flora and is expected to become infected and progress to deep infection and therefore fail. However, despite frequent colonization of the skin penetration site by potentially virulent bacteria, only few infections leading to implant removal have occurred [6]. The Osseointegrated Prostheses for the Rehabilitation of Amputees (OPRA; Integrum AB, Mölndal, Sweden) implant system has been used in transfemoral amputees for more than 20 years with recently reported cumulative success rate at 2-year followup of 92% in a prospective study in 51 patients [2]. These amputees have increased ROM in the hip and better sitting comfort compared to socket prostheses [3]. Radiostereometric analysis in transfemoral amputees indicated stable fixation of the implant and periprosthetic bone remodeling similar to that seen around uncemented hip stems [5]. The radiologic changes were consistent with stress shielding and included endosteal and distal bone resorption, cortical thinning, cancellization, and proximal trabecular buttressing (Table 1). In short-term studies, this periprosthetic bone remodeling has not compromised the stability of the implant in transfemoral amputees but its long-term clinical relevance is still unknown. Although the biomechanics differ between a residual femur and a residual humerus, similar findings should be expected in the radiographs of transhumeral amputees. However, to our knowledge, there still are no published data on implant survival, adverse events and radiologic signs of osseointegration, and adaptive bone remodeling in transhumeral amputees treated with this method.

**Table 1:** Definitions, adverse events and their severity, and radiologic changes

Adverse event	Definition
Superficial infection of the skin penetration site	Clinical signs of infection (redness, swelling, purulent discharge with positive bacterial cultures from the skin/abutment interface) necessitating the use of local, oral, and/or IV antibiotics
Skin reactions at the skin penetration site	Color change such as purpleness or redness, serous discharge, or the presence of a granulation ring; the latter is a ring of granulation tissue covered by epithelium that surrounds the abutment (Fig. 3)
Deep implant infection	Infection in the intramedullary canal proximally to the fixture, presenting with pain and swelling of the residual arm as well as positive intramedullary bacterial cultures
Incomplete distal fracture at S1 surgery	Incomplete fracture or erosion of the distal cortical bone while reaming or introducing the fixture in the form of a spiral fracture or a partial bone defect that does not compromise the fixture's primary stability
Defect of the bony canal at S2 surgery	Limited loss of the wall of the bony canal, which occurs during drilling for the introduction of the abutment at S2 surgery
Partial skin flap necrosis	Insufficient viability of the skin at the skin penetration site during the first weeks after S2 surgery
*Severity of adverse event*	*Definition*
Mild	Easily tolerated by the by the amputee
Moderate	Causes sufficient discomfort to interfere with daily activities
Severe	Causes hospitalization and/or surgery
*Radiologic changes*	*Definition*
Endosteal bone resorption	Resorption of endosteal bone around the threads of the fixture (Zones 1–12; Fig. 4)
Distal bone resorption	Resorption of the distal bone causing exposure of the fixture (Zones A, B, C, and D; Fig. 4)
Cancellization	Increase in the porosity of the cortex surrounding the fixture
Cortical thinning	Decrease of the width of the cortex around the fixture
Proximal trabecular buttressing	Increase in the density of the cortical and/or trabecular bone at the proximal end of the fixture

S1 = first surgery; S2 = second surgery; IV = intravenous.

We therefore sought to evaluate 2- and 5-year implant survival, adverse events, and radiologic signs of osseointegration and bone remodeling in transhumeral amputees treated with osseointegrated prostheses.

## Patients and Methods

The study was conducted as a retrospective case series.

Between 1995 and 2010, we performed 18 primary osseointegrated percutaneous implants in 18 transhumeral amputees; of those, two patients underwent implant revision as a result of early (< 2 years) fixture loosening and one patient had his abutment permanently removed as a result of shoulder osteoarthritis and subsequent shoulder arthrodesis. Of the initial number of transhumeral amputees, 16 patients were available for followup at a minimum of 2 years and 13 patients at 5 years (median, 8 years; range, 2–19 years). This group includes all transhumeral amputees who have received osseointegrated prostheses in our center and represents approximately 20% of the all transhumeral amputees we evaluated for potential osseointegration during that time; general indications for this approach included transhumeral amputation resulting from trauma or tumor, inability to wear or severe problems wearing a conventional socket prosthesis, eg, very short residual limb, and compliant patients.

The mean patient age at implantation was 42 years (range, 19–69 years); two were women and 16 men. The cause of amputation was either trauma (16) or malignant tumor (two) and the mean time interval from amputation to S1 was 9 years (range, 1.5–33 years). The study has been approved by the Swedish Regional Ethics Committee in Gothenburg.

**Fig. 2:**
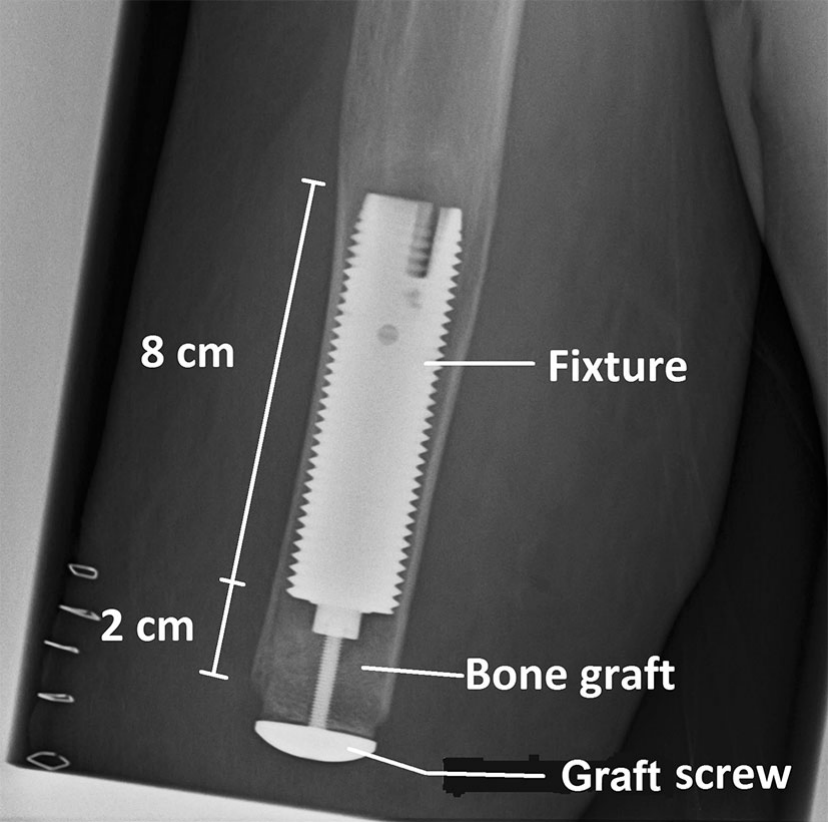
Direct postoperative radiograph is shown after S1. The fixture is countersunk by 2 cm into the medullary cavity and the residual space at its distal end is filled by autologous bone graft, which is held under compression by the so-called “graft screw.” At S2, the graft screw is removed and the healed bone graft is drilled to a diameter equal to the diameter of the abutment.

Transhumeral amputees referred to the Center of Orthopaedic Osseointegration in Gothenburg were assessed by a team consisting of an orthopaedic surgeon, an occupational therapist, a prosthetist, and a coordinator. Those who were eligible for osseointegration (approximately 20% of all transhumeral amputees) were scheduled for surgery. Briefly, the surgical technique at S1 consisted of reaming and tapping of the medullary cavity and insertion of the fixture, which was countersunk by 2 cm (Fig. 2). The residual space distally to the fixture was packed with autologous bone graft to keep the fixture away from the future skin penetration site and to create a bone stock that would prevent distal bone resorption from exposing the fixture and increase the contact area between bone and skin at S2. The wound was then closed. Postoperatively the patients were allowed to wear their socket prosthesis and after 4 to 6 months, they underwent S2. At S2 the distal bone was drilled and the abutment was inserted. All muscles were shortened and firmly attached to the periosteum of the humerus. A skin flap was raised and the skin penetration site was created and firmly attached to the distal end of the humerus. Six weeks after S2, a short training prosthesis was attached to the abutment and rehabilitation was started by successively increasing loading until they were able to wear a long prosthesis [4]. The followup protocol included clinical examination by an orthopaedic surgeon and an occupational therapist at the outpatient clinic with plain radiographic examination after S1 and S2 and 6 months and 1, 2, 3, 5, 7, 10, 13, and 15 years after S2.

**Fig. 3A–C:**
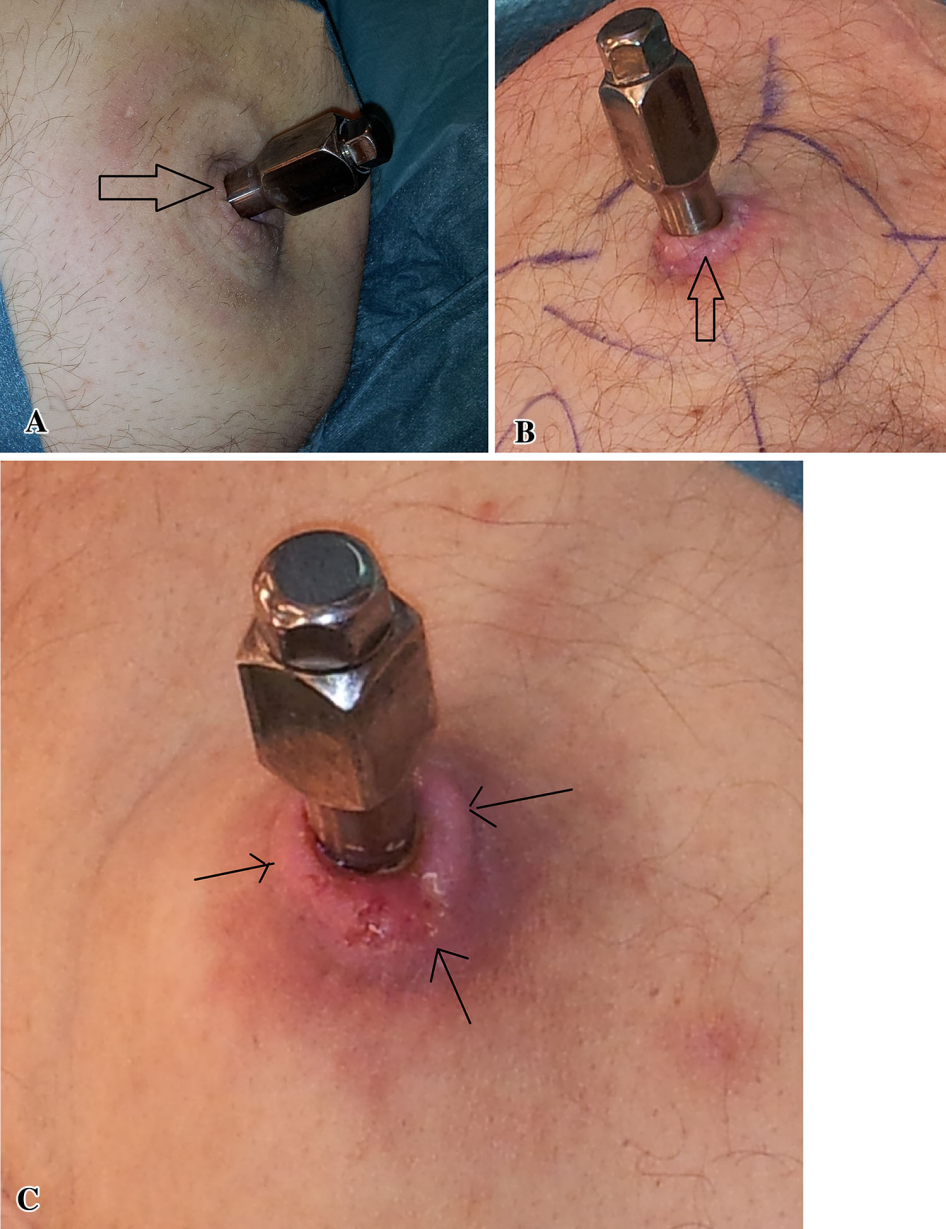
Skin reactions of the skin penetration site are shown. Changes in the skin color included purpleness (**A**) or redness (**B**). The skin around the abutment is elevated by underlying hypertrophic granulation tissue forming the “granulation ring” (**C**, arrows); purpleness and some serous secretion are also evident (**C**).

**Fig. 4A–B:**
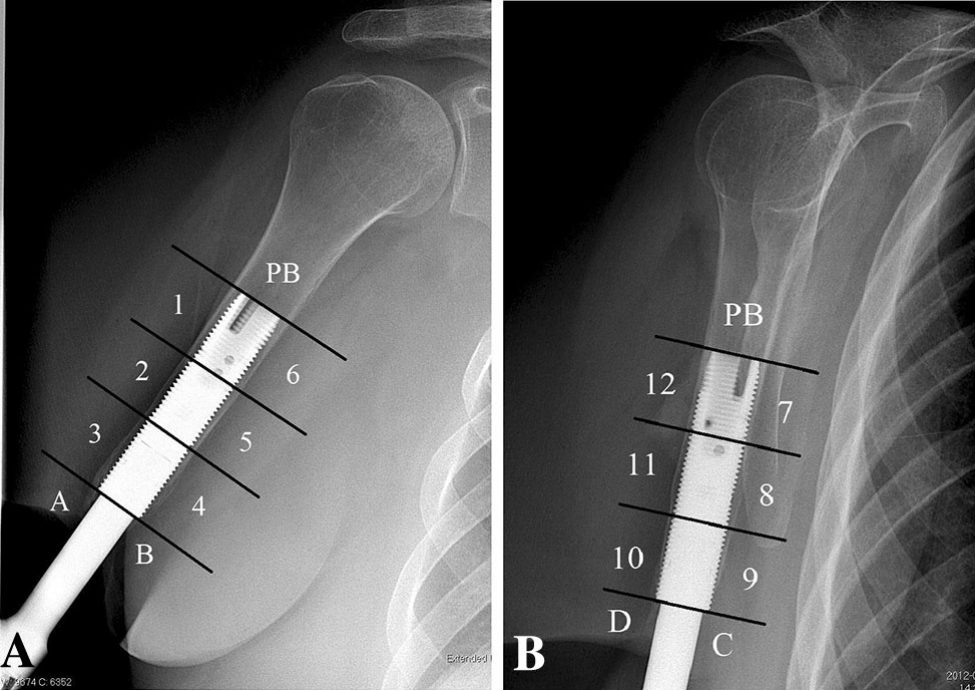
These AP (**A**) and lateral (**B**) views show the 12 zones (1–12) around the fixture and the four zones at the distal bone (**A**–**D**) as well as the bone proximally to the fixture (PB).

**Fig. 5A–B:**
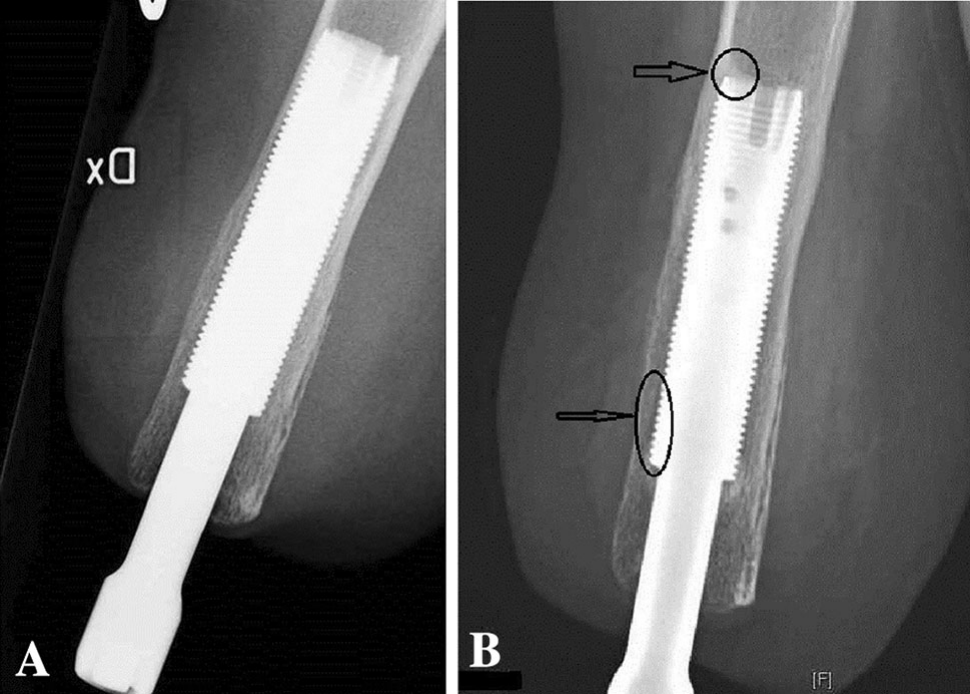
This 5-year postoperative radiograph (**B**) shows a well-fixed fixture with signs of endosteal bone resorption (lower arrow) and proximal trabecular buttressing (upper arrow). Comparison is made to the S2 postoperative radiograph (**A**).

**Fig. 6A–B:**
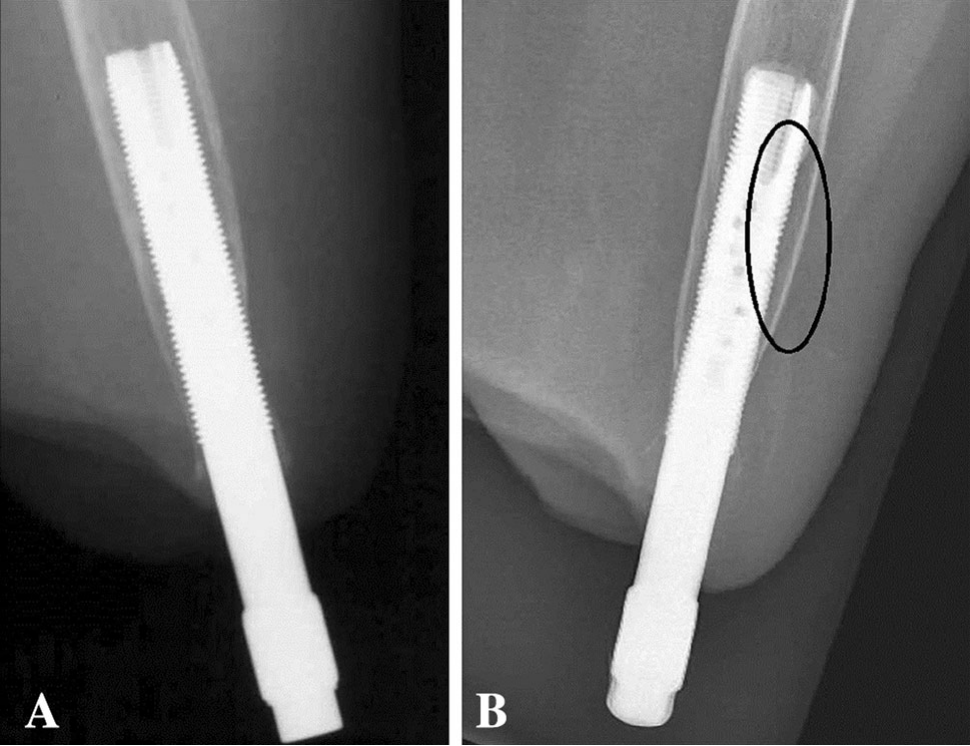
A postoperative radiograph at S2 is shown (**A**). Fifteen years later, cancellization is evident (**B**). This was one of the first patients who received an osseointegrated arm prosthesis. The fixture was custom-made and the technique of countersinking the fixture by 2 cm in the medullary cavity was not yet developed. This is why the distal bone looks as if it has already been resorbed at S2 (**A**).

**Fig. 7A–B:**
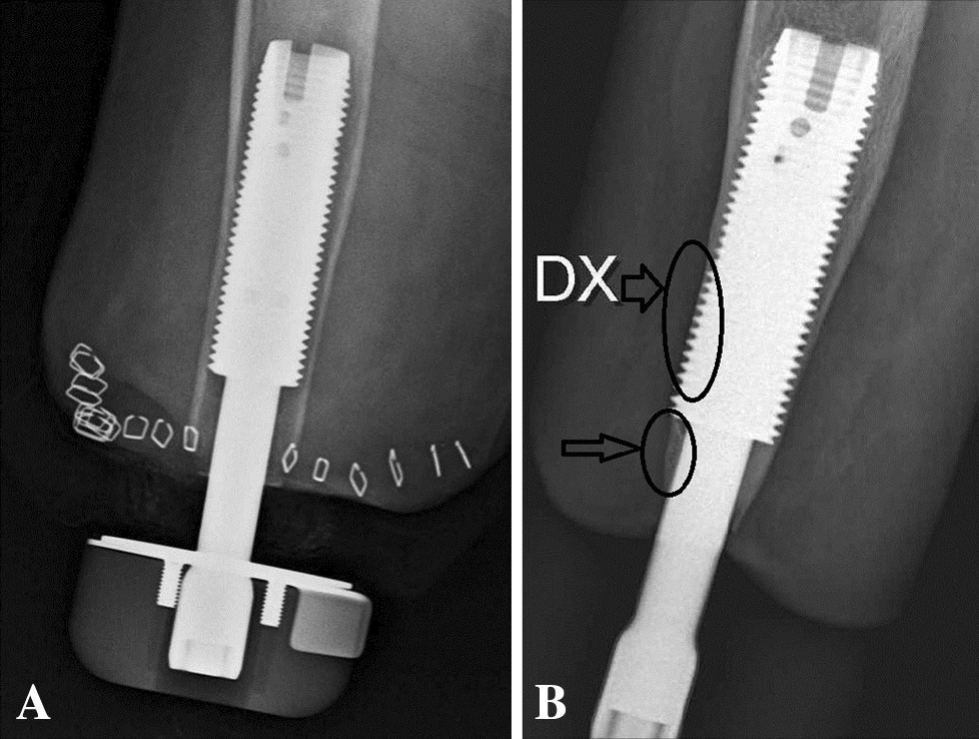
This radiograph at 3-year followup (**B**) shows signs of cortical thinning in the distal third of the fixture (upper arrow) and distal bone resorption at the bone canal distally to the fixture (lower arrow). Some proximal buttressing is also evident at the proximal third of the fixture. Comparison is made to the S2 postoperative radiograph (**A**).

The cumulative implant survival at each followup was measured as the number of implants that had not failed divided by the number of implants that could have failed since S1 (Table 2). Removal of the fixture or permanent removal of any implant component for any reason was considered as the endpoint for implant failure. For the study of adverse events and radiologic signs of bone remodeling, the medical charts and plain radiographs were examined by one observer (GT). The adverse events were divided into six categories according to the type of event: superficial infections of the skin penetration site, deep infections, skin reactions of the skin penetration site (Fig. 3), incomplete distal fractures at S1, defect of the bony canal at S2, and avascular skin flap necrosis (Table 1). Moreover, they were categorized according to their severity into mild, moderate, and severe (Table 1). The 2- and 5-year incidence was calculated for each adverse event where applicable. The plain radiographs included an AP and a lateral view of the residual arm perpendicular to the fixture. The bone around the fixture was divided into six zones and the distal bone into two zones on each view as previously done in transfemoral amputees with osseointegrated prostheses [5] (Fig. 4). The radiographs were examined for signs of endosteal bone resorption (Fig. 5), cancellization (Fig. 6), and cortical thinning (Fig. 7). The bone proximal to the fixture was examined for signs of trabecular buttressing (Fig. 5) and around the abutment for signs of distal bone resorption (Fig. 7). The same terminology and definitions were used as in transfemoral amputees for the results to be comparable (Table 1). The postoperative radiographs after S2 were used as a reference and were compared to the radiographs at each followup. The assessment of the radiographs was qualitative and each zone was either positive or negative for the mentioned radiological changes. This part of the study included both primary and revised implants (total of 20). In two patients the postoperative S2 radiographs were missing and the 6-month postoperative radiographs were used as a reference instead. Apart from these two radiographs, 10 more radiographs were missing. Those radiographs were counted as unchanged in relation to the previous radiographs.

**Table 2:** Implant survival at each followup

Followup (years)	Implants at risk for failure since the first surgery (could have failed)	Implants that have not failed since the first surgery	Implants that have failed since the first surgery	Survival (%)
0 (Stage 1)	18	18	0	100
0.5 (Stage 2)	18	18	0	100
1	18	16	2	89
2	18	15	3	83^*^
3	17	14	3	82
5	15	12	3	80^*^
7	11	8	3	
10	8	5	3	
13	6	3	3	
15	4	1	3	

^*^Note that the survival rate appears to drop between 2- and 5-year followup despite that no new implant failure has occurred during that time interval because there were three less implants available at 5 years than at 2 years.

## Results

### Implant Survival

The implant’s cumulative survival rate at 2 and 5 years (post-S1) was 83% and 80%, respectively (Table 2). Three patients had implant failure. In two patients the fixture failed as a result of loosening within 2 years from S1. Both patients underwent two-stage revision surgery. Intraoperative cultures were positive for *Staphylococcus* *aureus* and coagulase-negative staphylococci. After implant removal, the patients received antibiotics (clindamycin and rifampicin) and negative intramedullary cultures were secured before reimplantation. One of the two patients had a second fixture loosening; this patient underwent fixture removal and closure of the skin penetration site and is now not using any kind of prosthesis because the patient was already unsatisfied with socket prostheses before undergoing osseointegration. Intraoperative cultures at fixture removal were negative. The other patient has a well-fixed revised fixture up to date (6 years). In these two patients, the primary stability of the fixture at the first S1 was reported by the surgeon as unsatisfactory, meaning that the torque would not increase while installing the fixture deeper. All three fixture loosenings occurred within 2 years after implantation. There were no clinical signs of infection and the only symptom was pain at loading. The radiographs were unchanged since S1. No late fixture loosening has been observed. A third patient developed glenohumeral osteoarthritis in the ipsilateral shoulder and underwent shoulder arthrodesis. He was unable to wear any kind of prosthesis because of persistent shoulder pain and was treated with permanent removal of the abutment and closure of the skin penetration site leaving the osseointegrated fixture in the humerus. Although the reason for failure was not related to the implant, this implant was counted as failed because it could no longer serve as an anchoring point for prosthesis.

### Adverse Events

A total of 43 adverse events were recorded. Twenty-one (49%) were mild, 16 (37%) moderate, and six (14%) severe. Superficial infections of the skin penetration site accounted for 35% (15 infections) of all adverse events and were encountered in five patients. Their 2- and 5-year incidence was 19% (three of 16) and 38% (five of 13), respectively. Their treatment included surgical revision of the skin penetration site, local mechanical cleaning, local or oral antibiotics, and restriction of soft tissue mobility by using a silicone liner (Table 3). The duration of antibiotic treatment varied from 2 to 6 weeks. Although recurrences were common, in three of five patients, the infections healed uneventfully. In the remaining two patients, the infections healed but they developed increased soft tissue mobility at the skin penetration site. All five patients are still able to use their prosthesis. The most common pathogen was *S aureus* (three of five). Deep implant infection occurred in one patient 3½ years after S1 and without any history of superficial infection. It presented with mild signs of infection such as pain and tenderness in the residual arm, limited redness and discrete discharge from the skin penetration site, and periosteal reaction on the radiographs. Histology was consistent with osteomyelitis and cultures were positive for *Escherichia coli*. The patient was treated with oral antibiotics for 3 months, which resulted in complete regression of the infection and the patient being able to use the prosthetic arm again.

**Table 3:** Superficial infections at the skin penetration site: number of relapses, treatment, and clinical outcome

Patient number	Time of first infection^*^ (months)	Number of relapses	Number of relapses per year	Treatment	Outcome
1	4	6	2	3 surgical débridements of the SPS + 1 surgical revision of the SPS + antibiotics	Partial loss of skin attachment at SPS. Uses prosthesis
2	14	3	0.85	Antibiotics	Free of infection for 3 years
3	38	1	1	Antibiotics	Free of infection for 9 years
4	5	0		Surgical irrigation of the SPS + antibiotics	Increased mobility of soft tissues around the SPS; intermittent discharge; uses prosthesis
5	38	0		Antibiotics	Free of infection for 6 years

^*^Since second surgery; SPS = skin penetration site.

Skin reactions of the skin penetration site accounted for 19% of all adverse events and were noticed in eight patients. Their 2- and 5-year incidence was 38% (six of 16) and 62% (eight of 13), respectively. They were treated with clinical observation alone or local nonsurgical cleaning and chemical cauterization (AgNO_3_) and all but one resolved allowing the patients to use their prostheses (Table 4).

**Table 4:** Skin reactions of the skin penetration site: treatment and clinical outcome

Patient number	Time of first skin reaction^*^ (months)	Treatment	Outcome
1	60	Observation	Resolved
2	7	Observation	Persistent secretion, limited prosthetic use
3	50	Cauterization (AgNO_3_)	Resolved
4	16	Soft tissue supporting pad	Resolved
5	3.5	Cauterization	Resolved
6	6	Local nonsurgical cleaning	Resolved
7	12	Local nonsurgical cleaning	Resolved
8	17	Observation	Resolved

^*^Since second surgery.

Incomplete distal fracture of the residual bone at S1 accounted for 19% (eight fractures) of all adverse events and occurred in 44% (eight of 18) of S1 performed. In six fractures, no special treatment was conducted. One fracture was treated with autologous bone transplantation and another one with only modified passive rehabilitation between S1 and S2. All fractures were not evident on the radiographs 6 months postoperatively and S2 was performed as scheduled. However, one implant failed 2 years postoperatively. No periprosthetic fracture after S1 has occurred up to date. Adverse events at S2 included a limited defect of the bony canal (three) that occurred while drilling for the abutment and partial skin flap necrosis (three). Bone defects healed uneventfully and partial skin flap necroses healed within 2 to 4 weeks after skin débridement and treatment with oral antibiotics.

Apart from the mentioned adverse events, three amputees had phantom pain in their arm. In one of those, a neuroma was identified and removed during S2 leading to pain relief, whereas in the remaining two, the pain was persistent despite treatment with amitriptyline and gabapentine. A fourth amputee sustained a collum chirurgicum fracture after falling, which healed with nonsurgical treatment.

### Radiologic Findings

**Fig. 8:**
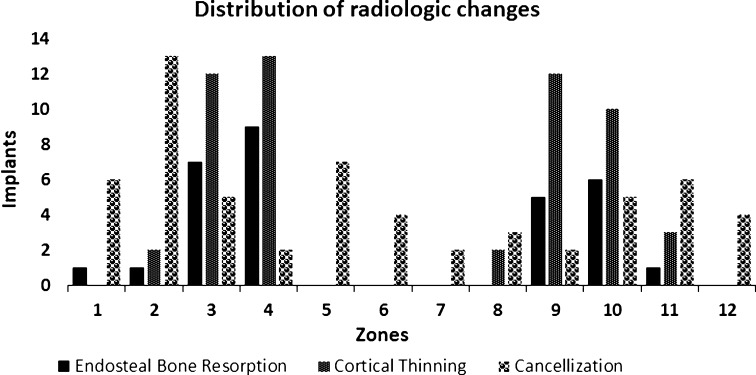
This chart shows the distribution of endosteal bone resorption, cortical thinning, and cancellization in the 12 zones around the fixture. Endosteal bone resorption and cortical thinning were observed mainly in the distal third of the bone-fixture interface (Zones 3, 4, 9, and 10), whereas cancellization was observed mainly in its middle third (Zones 2, 5, 8, and 11).

The most common radiologic finding was proximal trabecular buttressing (10 of 20 implants) followed by endosteal bone resorption and cancellization (seven of 20), cortical thinning (five of 20), and distal bone resorption (three of 20). Proximal buttressing became more common with time with its frequency increasing from five of 16 implants at 2 years to six of 13 implants at 5 years. Cancellization had also an increasing trend from three of 16 to three of 13. Cortical thinning had a low frequency at 2 and 5 years (four of 16 and two of 13, respectively) and endosteal bone resorption was observed in up to three implants at each followup. Distal bone resorption was observed once at 2 years and twice at 5 years. Moreover, it was limited and never exposed the thread of the fixture. Cancellization was distributed quite evenly among the zones around the fixture showing a slight preference for its middle third, whereas near bone resorption and cortical thinning were evident mostly around its distal third (Fig. 8).

## Discussion

The implementation of osseointegration for the rehabilitation of amputees challenges the basic principle of implant surgery stating that implants must be sufficiently covered by soft tissues to avoid infection. Although there is > 20 years of experience of percutaneous bone-anchored implants in transfemoral amputees [2], this method is still met with skepticism in the orthopaedic community. This report is the first of which we are aware on infectious and other adverse events in transhumeral amputees; we also sought to provide data on the 2- and 5-year implant survival and to comment on osseointegration and adaptive bone remodeling around these implants.

This study has certain limitations. The number of patients (18) is low and the study was retrospective. The patient cohort was selected by a multidisciplinary team (orthopaedic surgeon, occupational therapist, prosthetist, coordinator) based on the patient’s reason for amputation (trauma or tumor), wish for better function, and estimated compliance without randomization and represents approximately 20% of all transhumeral amputees that are referred to our center. Therefore, highly motivated patients with good compliance were more likely to be selected for osseointegration. However, the observer (GT) who reviewed the medical charts and plain radiographs was not part of this multidisciplinary team or active in the treatment and followup of the amputees. Moreover, no comparison was made between the osseointegration cohort and amputees with socket arm prostheses; also, the study did not include any patient-reported outcomes for pain, function, and prosthetic use, which makes it difficult to make any conclusions about the superiority of one or the other method. In some instances, the patients missed their followup appointment resulting in potential adverse events being registered at the next followup. In these instances the exact time when the adverse event had occurred was not registered in the medical charts. Because a part of the implant is in direct contact with the outer environment and therefore contaminated by bacteria, it is difficult to draw a distinct line between skin reactions as a result of inflammation of the soft tissues of the skin penetration site and manifest bacterial superficial infections. Clinical signs of inflammation, positive bacterial cultures, and antibiotic treatment given were used as criteria for infection trying to distinguish infection from inflammation. Bacterial contamination has however previously been found in half of asymptomatic patients with an osseointegrated percutaneous implant [6]. Bacteria may be commensals, mutualistic, or pathogenic and potential “pathogens” may or may not actually produce infection. Presumably some inflammatory skin reactions have been registered as superficial infections and vice versa. All radiographs were not available for examination and moreover the assessment of radiologic changes was made by one observer (GT) without calculating intraobserver error.

This implant system had a 2- and 5-year survival rate of 83% and 80%, respectively, in transhumeral amputees, which appears lower than the 2-year survival rate (92%) of the same implant system in transfemoral amputees in the OPRA study [2]. We believe that this difference can be explained by the higher experience of our center in transfemoral amputees and that the use of custom-designed components can increase the risk of not having optimal primary stability at implant insertion. In contrast to endoprostheses such as hip and knee prostheses, aseptic loosening cannot practically be diagnosed because the system is open to the outer environment and any failed osseiontegration inevitably leads to contamination of the bone-fixture interface. The importance of good primary stability of the fixture at S1 is highlighted because poor primary stability could compromise osseointegration and was reported in two implant failures. No mechanical problems of the implant systems occurred, in contrast to transfemoral amputees in whom bending or fracture of the abutment or the abutment screw has been reported [2].

Superficial infections of the skin penetration site were the most common adverse event in transhumeral amputees. At 2 years, three of 16 (19%) and at 5 years five of 13 (38%) patients had developed at least one superficial infection. Transfemoral amputees had a frequency of infection of 58% (28 of 48 patients at 2 years), which might be the result of less mobility of the soft tissues in transhumeral amputees [2]. Nonsurgical treatment or minor skin revisions was sufficient in all superficial infections, but the treatment needs to be standardized. None of the superficial infections progressed to a deep implant infection or caused implant revision during the study period. The only deep implant infection occurred late (3.5 years after S1) in contrast to transfemoral amputees in whom deep infections were reported early in the postoperative period [2]. Skin reactions represent an inflammatory status of the skin penetration site that may or may not lead to a superficial infection. In our study, only three of eight patients with skin reactions of the skin penetration site developed a superficial infection after 8 to 30 months, indicating that skin reactions alone are not a sufficient factor for the development of superficial infections. A classification system of the skin reactions of the skin penetration site with a predictive value for the risk of development of superficial infections would be useful. Skin motion around the abutment is believed to be a predisposing factor for superficial infections and/or skin reactions of the skin penetration site. Our method is based on firmly attaching the skin onto the distal bone at S2 to minimize the risk for such adverse events. On the other hand, superficial infections, especially combined with distal bone resorption, could lead to loss of skin attachment and therefore increase the risk for new superficial infections of the skin penetration site. In our series loss of skin attachment was partial and occurred in one patient at 15-year followup after six relapses of superficial infections of the skin penetration site (Table 3). In this retrospective study, details of attachment of the skin penetration site were not possible to evaluate thoroughly. We hypothesize that a well-attached skin penetration site will lead to less draining and infectious complications but further studies are needed to explore this. Skin reactions and superficial infections of the skin penetration area were common but were not severe and were easily managed. These complications should be weighed against the common complaints of transhumeral amputees using a socket prosthesis, which include restricted shoulder ROM and discomfort because of warmth and excessive perspiration caused by the socket and the heavy harness. Prosthetic rejection has been reported between 23% and 26% [1] in this group, whereas in our cohort, 16 of 18 patients still use their osseointegrated prosthesis. The absence of a socket and harness in patients with osseointegrated arm prostheses should eliminate these problems reported by almost all conventional socket arm prosthesis users.

The residual bone around the implant in transhumeral amputees showed radiologic changes similar to those in transfemoral amputees although with some differences. Distal bone resorption in the humerus occurred to a much lesser extent than in the femur and did not result in exposure of the fixture. Proximal buttressing, which was the most common radiologic change in the humerus, also appeared differently and looked rather like uniform thickening of the bone at the proximal third and above the fixture than triangular areas as observed in the transfemoral amputees [5]. This may be the result of the different forces that act on these areas, because the residual femur is exposed for mainly compressive forces and bending moments (walking), whereas the residual humerus is exposed for mainly tensile forces and bending moments (lifting). The latter put more loading on the distal bone and less on the proximal bone in transhumeral amputees compared with transfemoral. Although the presence of proximal buttressing and distal bone resorption can be explained by Wolff’s law as bone remodeling consistent with stress shielding, the clinical relevance of cancellization and cortical thinning is uncertain because long-term studies are not available. They could theoretically represent risk factors for periprosthetic fracture in years to come. Endosteal bone resorption is also a potential threat that could compromise the stability of the fixture if extending more proximally than the distal third of the fixture where it has so far been observed. Bone remodeling at the bone-fixture interface has so far not affected the osseointegration of the fixture because no late loosening or periprosthetic fracture has been reported.

To our knowledge, this is the first study on implant survival, adverse events, and radiologic signs of bone remodeling in transhumeral amputees treated with an osseointegrated percutaneous implant, reporting up to 19 years followup. We found an implant survivorship of 83% at 2 years and 80% at 5 years. The frequency of skin reactions and infectious complications related to the skin penetration site was relatively high (38% at 5 years), although most of them were not serious and were easily managed with nonoperative treatment. We also found a number of radiological changes that need to be followed over time because some of them have uncertain clinical relevance. Even so, we believe osseointegrated arm prostheses are a potentially attractive alternative to conventional socket prosthesis that should be considered, especially in very high transhumeral amputations in which adequate suspension of a socket prosthesis is difficult. Osseointegrated arm prostheses have so far only been used in amputations resulting from either trauma or tumor. It is uncertain whether the implant has a similar survivorship in amputations resulting from vascular disease. Our approach could theoretically provide transhumeral amputees with better comfort and a greater shoulder ROM than socket prostheses. Comparative studies are needed to support its potential superiority.
